# First Clinical Applications of a High-Definition Three-Dimensional Exoscope in Pediatric Neurosurgery

**DOI:** 10.7759/cureus.2108

**Published:** 2018-01-24

**Authors:** Thomas Beez, Christopher Munoz-Bendix, Kerim Beseoglu, Hans-Jakob Steiger, Sebastian A Ahmadi

**Affiliations:** 1 Department of Neurosurgery, Medical Faculty, Heinrich-Heine-University

**Keywords:** pediatric neurosurgery, microsurgery, exoscope, microscope

## Abstract

The ideal visualization tools in microneurosurgery should provide magnification, illumination, wide fields of view, ergonomics, and unobstructed access to the surgical field. The operative microscope was the predominant innovation in modern neurosurgery. Recently, a high-definition three-dimensional (3D) exoscope was developed. We describe the first applications in pediatric neurosurgery. The VITOM 3D exoscope (Karl Storz GmbH, Tuttlingen, Germany) was used in pediatric microneurosurgical operations, along with an OPMI PENTERO operative microscope (Carl Zeiss AG, Jena, Germany). Experiences were retrospectively evaluated with five-level Likert items regarding ease of preparation, image definition, magnification, illumination, field of view, ergonomics, accessibility of the surgical field, and general user-friendliness. Three operations were performed: supratentorial open biopsy in the supine position, infratentorial brain tumor resection in the park bench position, and myelomeningocele closure in the prone position. While preparation and image definition were rated equal for microscope and exoscope, the microscope’s field of view, illumination, and user-friendliness were considered superior, while the advantages of the exoscope were seen in ergonomics and the accessibility of the surgical field. No complications attributed to visualization mode occurred. In our experience, the VITOM 3D exoscope is an innovative visualization tool with advantages over the microscope in ergonomics and the accessibility of the surgical field. However, improvements were deemed necessary with regard to field of view, illumination, and user-friendliness. While the debate of a “perfect” visualization modality is influenced by personal preference, this novel visualization device has the potential to become a valuable tool in the neurosurgeon’s armamentarium.

## Introduction

Visualization tools in microneurosurgery should provide magnification, illumination, wide fields of view, ergonomics, and unobstructed surgical field access. The operating microscope was introduced in the 1960s and transformed procedures and outcomes [1]. Modern microscopes offer high magnification, extensive maneuverability, foldable co-observation tubes, high degrees of automation, flat screens for observers, and integrated advanced features, such as tumor fluorescence, intraoperative angiography, and head-up display neuronavigation. These features make modern microscopes even more versatile than those microscopes that revolutionized neurosurgery many decades ago [2]. However, with the emergence of 4K and 8K imaging (the so-called ultra-high definition), it is imaginable that further evolutions could be imminent. Ever smaller imaging sensors, virtual and augmented reality devices, and novel 3D interfaces could refine intraoperative visualization [3-4]. Recently, a high-definition 3D exoscope was developed with the attributes of being smaller and less expensive than microscopes and not confining surgeons to eyepieces. We describe the first clinical applications in pediatric neurosurgery.

## Technical report

The VITOM 3D exoscope (Karl Storz GmbH, Tuttlingen, Germany) was used in pediatric neurosurgery, with initial onsite support and training by the manufacturer. The system consists of two 4K (4096 x 2160 pixels) sensors, providing a large, scrollable field of view, a quarter of which is displayed as a full high definition (HD) (1080p resolution) image on a 32-inch active 3D screen. This allows for passive (polarized) and, consequently, light 3D glasses or overlays for those wearing corrective glasses. We used it in addition to an OPMI PENTERO microscope (Carl Zeiss AG, Jena, Germany) and switched between both modalities at the surgeon’s discretion. To share our experiences with the neurosurgical community, the opinions of the surgeon and assistant were retrospectively evaluated with questionnaires with five-level Likert items (from 1 = very good to 5 = very insufficient) regarding preparation (including set-up, balancing, positioning, and draping), image definition, magnification, illumination, field of view, working environment ergonomics, accessibility of the surgical field, and user-friendliness (including using zoom and focus, changing the position of the visual field). We performed separate analyses for the surgeon and assistant to assess differences in perception as well as a pooled analysis to give median scores for overall comparison.

**Figure 1:**
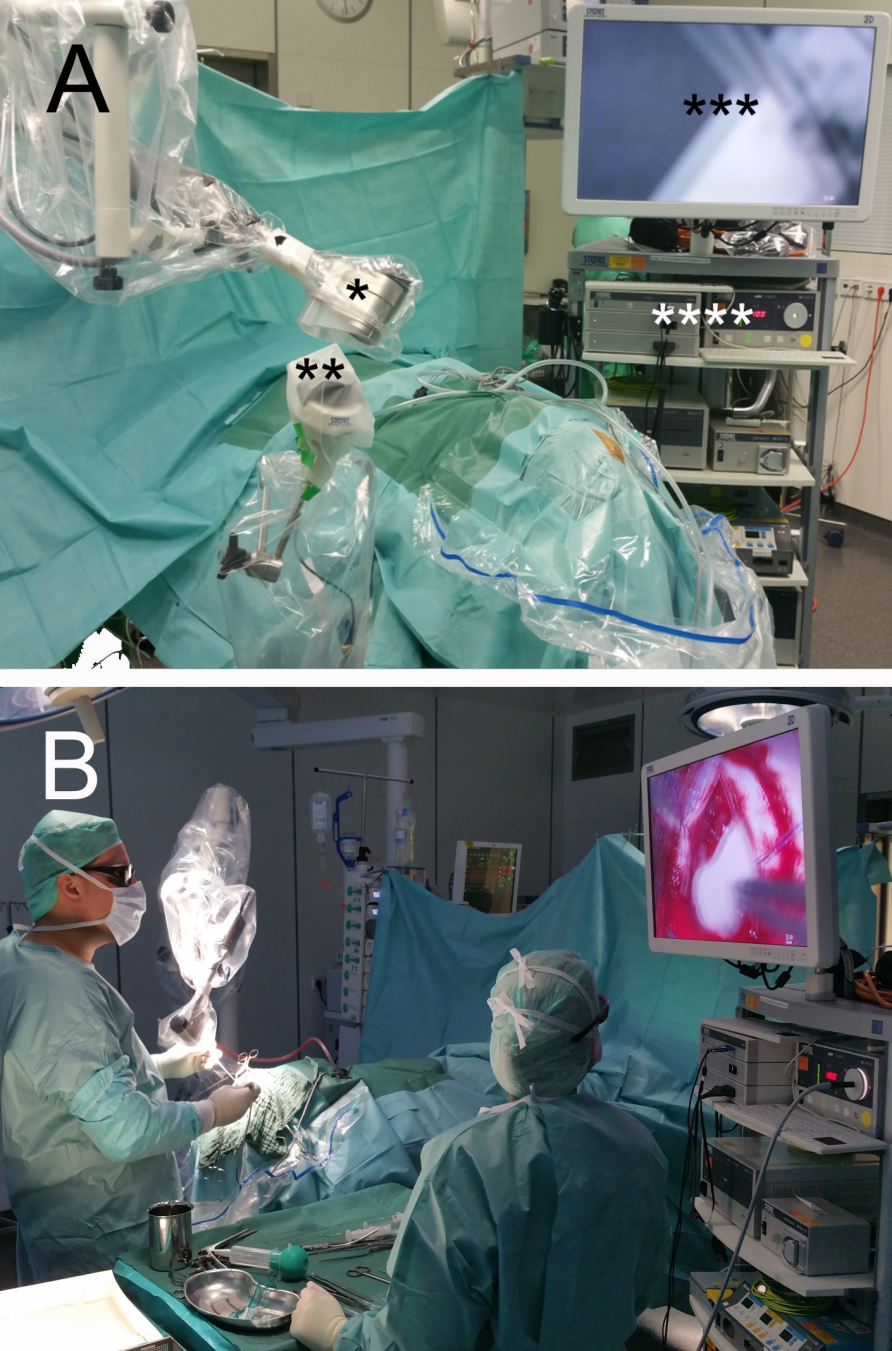
Operating room set-up and exoscope during surgery Operating room set-up (A) with * = VITOM 3D exoscope attached to holding arm (Karl Storz GmbH, Tuttlingen, Germany), ** = IMAGE 1 PILOT, *** = 3D monitor, and **** = IMAGE 1 camera system and cold light fountain. Exoscope during surgery (B), depicting the surgeon’s ergonomic posture.

We discuss the technical aspects and initial experiences in three consecutive pediatric operations using both exoscope and microscope, performed by four board-certified neurosurgeons (TB, CMB, KB, SAA): In a five-year-old boy, an open biopsy of a right frontal supratentorial lesion of unknown etiology was carried out in the supine position with rigid head fixation and neuronavigation. A 10-year-old girl underwent resection of the infratentorial pilocytic astrocytoma in the park bench position with rigid head fixation and intraoperative neuromonitoring. Finally, in a male neonate, lumbosacral myelomeningocele closure was performed in the prone position. Informed consent for the microsurgical operations was obtained from parents or guardians, but since the exoscope was used as a fully approved medical device in routine medical care without research intention, no separate consent was necessary. This is a purely technical retrospective report containing very limited and anonymized patient information. The operating room set-up for the exoscope is demonstrated in Figure 1. No complications attributed to visualization mode occurred.

A separate analysis (Table 1) of ratings by the surgeon and assistant revealed similar scores for preparation, magnification, and image definition. Illumination and user-friendliness were rated differently, with the surgeon’s exoscope ratings being worse due to an oversensitive main control button (IMAGE 1 PILOT, Karl Storz GmbH, Tuttlingen, Germany), leading to difficulties in achieving the desired image adjustments and due to the inferior illumination along deep corridors.

**Table 1:** Comparison of microscope and exoscope Results of category ratings by surgeon (S) and assistant (A) on a five-level Likert scale (1 = very good, 2 = good, 3 = neutral, 4 = insufficient, 5 = very insufficient).

	Preparation		Image definition		Magnification		Field of view		Illumination		Working environment ergonomics		Accessibility of the surgical field		User-friendliness	
	S	A	S	A	S	A	S	A	S	A	S	A	S	A	S	A
Supratentorial (frontal brain lesion, open biopsy)																
Microscope	2	2	1	1	1	1	1	2	1	1	2	2	1	2	1	2
Exoscope	2	2	1	1	2	2	1	2	2	2	1	1	1	1	3	2
Infratentorial (cerebellar tumor resection)																
Microscope	2	3	1	1	1	2	1	1	1	2	3	1	2	2	1	2
Exoscope	2	2	1	2	2	1	1	2	3	1	2	2	2	2	3	2
Spinal (repair of lumbosacral myelomeningocele)																
Microscope	2	2	1	1	1	1	1	2	1	1	1	2	1	2	1	2
Exoscope	2	2	1	1	2	2	1	2	2	3	1	3	1	3	3	2

**Figure 2:**
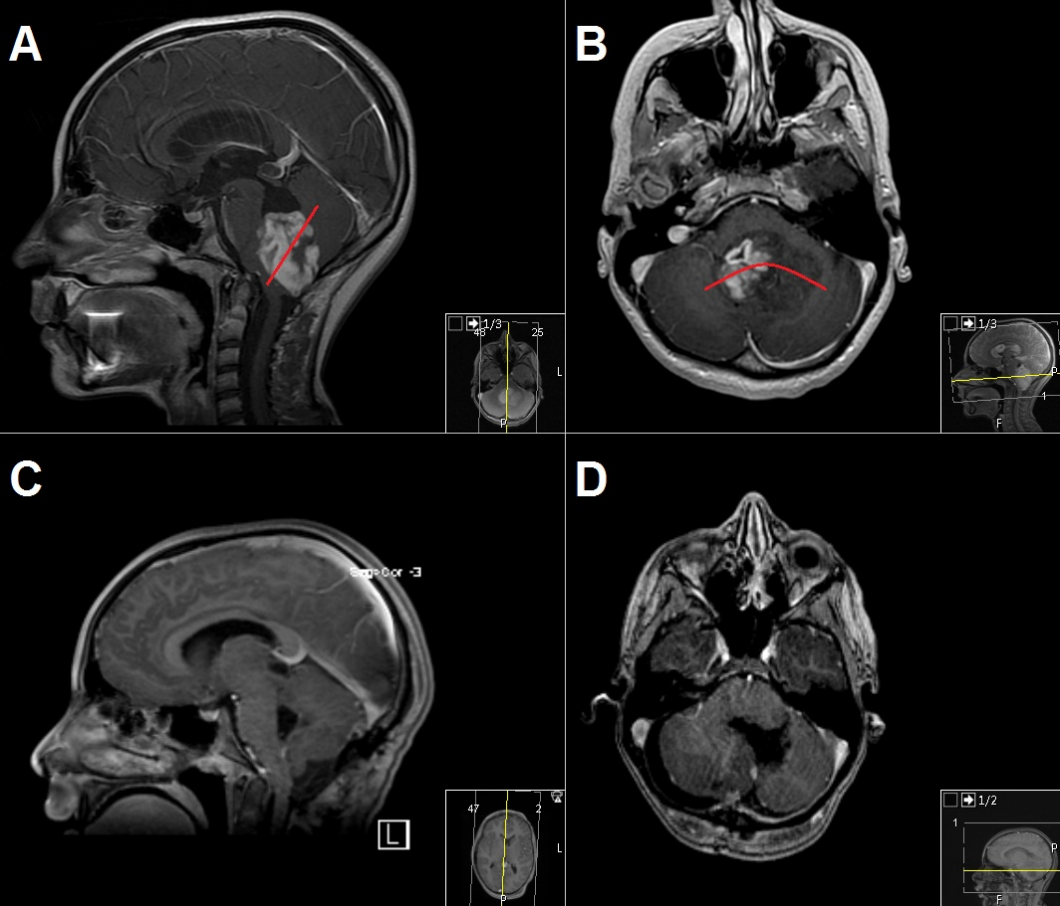
Illustrative magnetic resonance imaging of the case "Infratentorial (cerebellar tumor resection)" Preoperative (A and B) and postoperative (C and D) contrast-enhanced T1-weighted magnetic resonance imaging (MRI) sequences of a 10-year-old girl with infratentorial pilocytic astrocytoma. The red lines in 2A and 2B demonstrate the approximate limitation of the exoscope in the surgeon’s experience. Further resection of the deeper portions of the tumor was performed under a microscope.

Of note, the illumination was felt to be insufficient in the posterior fossa case as the dissection advanced, with a switch to the microscope required for the removal of the deeper portions of the tumor toward the fourth ventricle, as depicted in Figure 2. Additionally, the lack of lateral inclination of the exoscope’s axis of view was felt to be a limitation. Further differences between the surgeon and assistant were found concerning ergonomics and access to the surgical field, particularly for the posterior fossa and spinal cases. Wearing 3D goggles was not felt to put additional strain on the surgical team.

**Figure 3:**
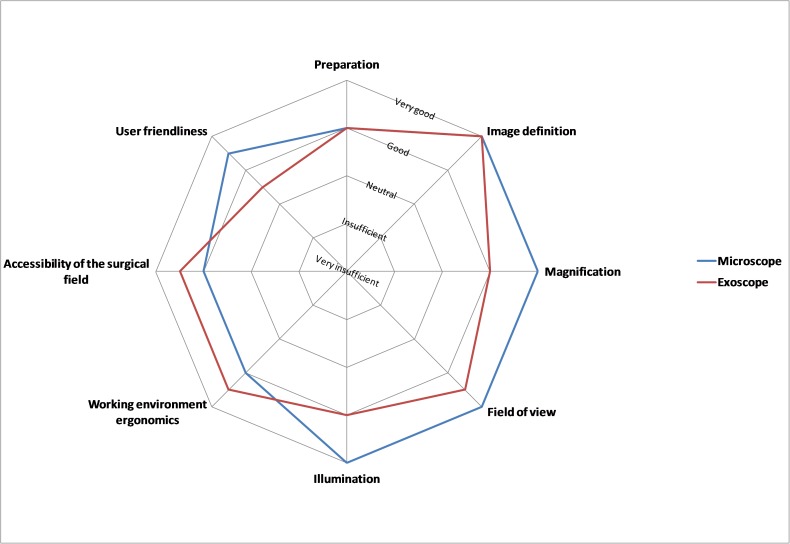
Results of the pooled analysis of surgeons' and assistants' questionnaires (median values)

A pooled analysis (Figure 3) demonstrated equal median ratings for microscope and exoscope for preparation and image definition. The microscope was rated superior for magnification, field of view, illumination, and user-friendliness. The exoscope was rated superior for ergonomics and the accessibility of the surgical field. The overall ratings (mean and standard deviation (SD)) for the microscope and exoscope showed a preference of the surgical team in favor of the microscope by tendency: 1.44 (SD 0.51) and 1.63 (SD 0.62) for the supratentorial lesion, 1.63 (SD 0.72) and 1.88 (SD 0.62) for the posterior fossa tumor, and 1.38 (SD 0.50) and 1.94 (SD 0.77) for the myelomeningocele, respectively. While not the focus of this analysis, scrub nurses reported an improved and more immersive experience, being previously confined to 2D-only screen visualizations of heavily magnified fields of view.

## Discussion

While preparation and image definition were rated equal for microscope and exoscope, the microscope’s field of view, illumination, and user-friendliness were considered superior, while the advantages of the exoscope were seen in ergonomics and the accessibility of the surgical field.

In previous publications on earlier exoscope models, the lack of stereopsis was a drawback [5-6]. This has been overcome by using two 4K sensors providing full HD 3D visualization. In our experience, this certainly enhances the exoscope, along with its large depth of field, although the potential advantages of these features could be strengthened further by more focused illumination. Similar to previous reports, the surgeon is no longer confined to eyepieces and can more freely arrange the layout of operating room equipment and personnel [7]. In our limited experience, the ergonomics of the exoscope workplace were rated differently by surgeon and assistant for different patient positions, probably reflecting limitations inherent to patient positioning or inexperience with ergonomically positioning the exoscope’s components for all team members. We assume that adaptation to the exoscope might be easier for neurosurgeons used to endoscopic surgery, as the exoscope carries many similarities to a neuroendoscopy working environment. Since most pediatric neurosurgeons perform a larger percentage of neuroendoscopy cases, the learning curve and potential for acceptance might be higher in this subgroup. Observers benefit from watching the operation on a high-definition 3D screen, improving the teaching and learning experience [8].

In our opinion, improvement is desirable for the exoscope’s main control button, which is highly sensitive and causes difficulties in achieving the desired image adjustments. Further refinement is possible by introducing automated lateral inclination of the exoscope’s axis of view instead of crude manual adjustments of the holding arm. This limitation was evident when trying to stop hemorrhage just below the overhanging cortical edge. With more focused light-emitting diode (LED) lighting, differences in illumination between microscopes and 3D exoscopes could likely be mitigated.

The limitations of this report are the small number of cases and the lack of a learning curve. The perceived advantages of the microscope, especially regarding user-friendliness, might be related to this. Nevertheless, we consider it worthwhile sharing our first applications of the exoscope with the neurosurgical community, i.e., potential buyers of this new commercially available tool.

## Conclusions

In our experience, the exoscope is an innovative visualization tool with advantages over the microscope in working environment ergonomics and the accessibility of the surgical field. However, improvements are desirable with regards to field of view, illumination, and user-friendliness. While the debate of a “perfect” visualization modality is influenced by personal preference, this technique has the potential to become a valuable tool in the neurosurgeon’s armamentarium. Whether the exoscope will decrease the surgeon’s fatigue or even allow for new techniques or better outcomes will be demonstrated over time, as more users report their experiences.

## References

[1] Uluç K, Kujoth GC, Başkaya MK (2009). Operating microscopes: past, present, and future. Neurosurg Focus.

[2] Zebian B, Vergani F, Lavrador JP (2017). Recent technological advances in pediatric brain tumor surgery. CNS Oncol.

[3] Heath MD, Cohen-Gadol AA (2012). Intraoperative stereoscopic 3D video imaging: pushing the boundaries of surgical visualisation and applications for neurosurgical education. Br J Neurosurg.

[4] Christopher LA, William A, Cohen-Gadol AA (2013). Future directions in 3-dimensional imaging and neurosurgery: stereoscopy and autostereoscopy. Neurosurgery.

[5] Mamelak AN, Danielpour M, Black KL, Hagike M, Berci G (2008). A high-definition exoscope system for neurosurgery and other microsurgical disciplines: preliminary report. Surg Innov.

[6] Mamelak AN, Nobuto T, Berci G (2010). Initial clinical experience with a high-definition exoscope system for microneurosurgery. Neurosurgery.

[7] Oertel JM, Burkhardt BW (2017). Vitom® -3D for exoscopic neurosurgery: initial experience in cranial and spinal procedures. World Neurosurg.

[8] Moisi MD, Hoang K, Tubbs RS (2017). Advancement of surgical visualization methods: comparison study between traditional microscopic surgery and a novel robotic optoelectronic visualization tool for spinal surgery. World Neurosurg.

